# Comparative Single-Cell Analysis of Different *E*. *coli* Expression Systems during Microfluidic Cultivation

**DOI:** 10.1371/journal.pone.0160711

**Published:** 2016-08-15

**Authors:** Dennis Binder, Christopher Probst, Alexander Grünberger, Fabienne Hilgers, Anita Loeschcke, Karl-Erich Jaeger, Dietrich Kohlheyer, Thomas Drepper

**Affiliations:** 1 Institute of Molecular Enzyme Technology, Heinrich-Heine-University Düsseldorf, Forschungszentrum Jülich, Jülich, Germany; 2 Institute of Bio- and Geosciences (IBG-1), Forschungszentrum Jülich, Jülich, Germany; New England Biolabs Inc, UNITED STATES

## Abstract

Recombinant protein production is mostly realized with large-scale cultivations and monitored at the level of the entire population. Detailed knowledge of cell-to-cell variations with respect to cellular growth and product formation is limited, even though phenotypic heterogeneity may distinctly hamper overall production yields, especially for toxic or difficult-to-express proteins. Unraveling phenotypic heterogeneity is thus a key aspect in understanding and optimizing recombinant protein production in biotechnology and synthetic biology. Here, microfluidic single-cell analysis serves as the method of choice to investigate and unmask population heterogeneities in a dynamic and spatiotemporal fashion. In this study, we report on comparative microfluidic single-cell analyses of commonly used *E*. *coli* expression systems to uncover system-inherent specifications in the synthetic M9CA growth medium. To this end, the P_T7lac_/LacI, the P_BAD_/AraC and the Pm/XylS system were systematically analyzed in order to gain detailed insights into variations of growth behavior and expression phenotypes and thus to uncover individual strengths and deficiencies at the single-cell level. Specifically, we evaluated the impact of different system-specific inducers, inducer concentrations as well as genetic modifications that affect inducer-uptake and regulation of target gene expression on responsiveness and phenotypic heterogeneity. Interestingly, the most frequently applied expression system based on *E*. *coli* strain BL21(DE3) clearly fell behind with respect to expression homogeneity and robustness of growth. Moreover, both the choice of inducer and the presence of inducer uptake systems proved crucial for phenotypic heterogeneity. Conclusively, microfluidic evaluation of different inducible *E*. *coli* expression systems and setups identified the modified *lacY*-deficient P_T7lac_/LacI as well as the Pm/XylS system with conventional *m*-toluic acid induction as key players for precise and robust triggering of bacterial gene expression in *E*. *coli* in a homogeneous fashion.

## Introduction

While in natural environments, cell-to-cell variations in gene expression and growth may prove beneficial and are considered as bet-hedging or division of labor strategies to enhance environmental adaptability within an isogenic bacterial population [1,2], such phenotypic heterogeneity is unfavorable in biotechnology and synthetic biology. Here, phenotypic homogeneity is needed to reliably predict and control target gene expression [3,4]. In this context, strength, velocity and tightness of gene expression responses seem essential for processes where, for instance, a general interconnection between biomass formation and product accumulation exists. Hence, expression systems should be critically evaluated down to single-cell level with respect to responsiveness, growth behavior and expression phenotype, to gain detailed insights into these processes and, subsequently, to yield a higher degree of control over target gene expression.

The last decades gave rise to several sophisticated inducible bacterial expression systems that were predominantly inspired by natural regulatory circuits. Mainly catabolic regulatory networks such as those for lactose, arabinose or benzoate utilization were employed as useful tools for heterologous gene expression [5–7]. These expression systems commonly consist of native or mutagenized promoters and a corresponding transcriptional regulator that represses, derepresses or activates target gene expression in the presence of a specific inducer that can enter the cell *via* an appropriate transport system or by passive diffusion.

**Fig 1:**
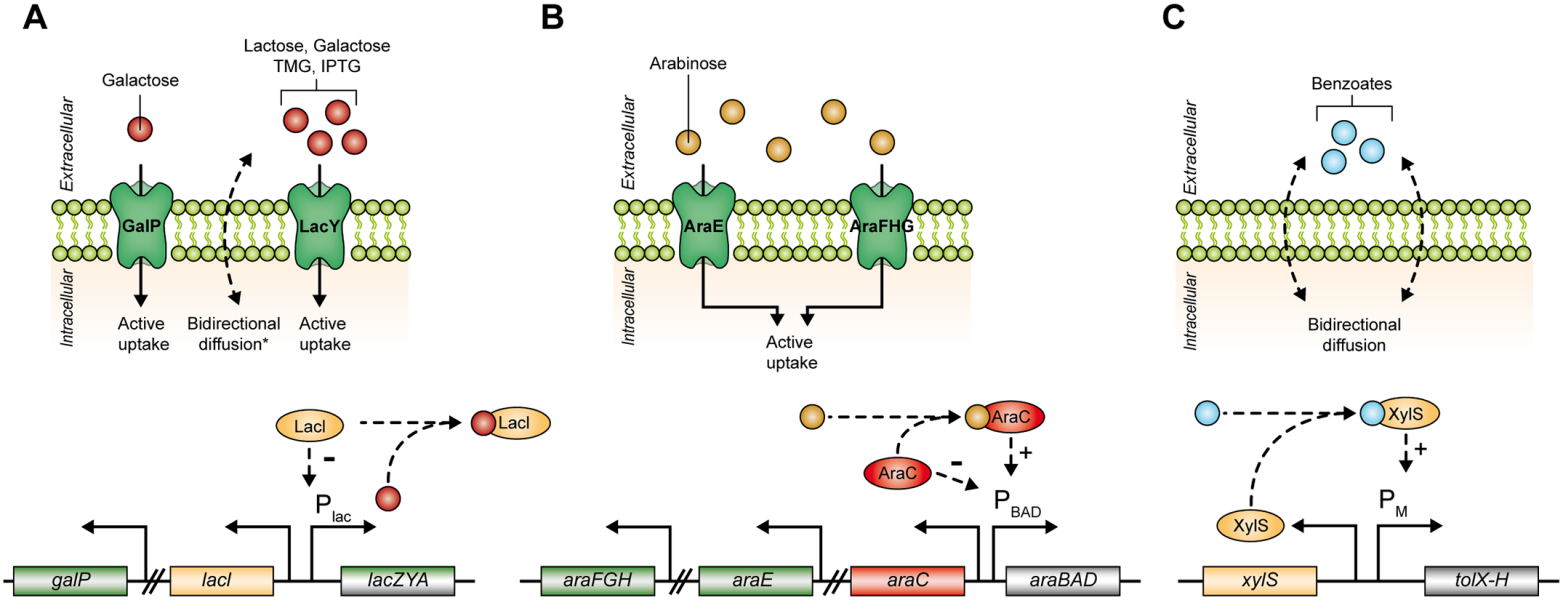
Simplified mechanisms of inducer uptake and regulation of target gene expression in common *E*. *coli* expression systems. (A) *Lac* based gene expression *via* natural (lactose, galactose) or synthetic (TMG, IPTG) inducers. Uptake basically occurs through GalP (mainly galactose) or LacY (all inducers) transport proteins and by passive diffusion (* only synthetic inducers TMG and IPTG). Inducer binding leads to the release of the LacI repressor from the P_lac_ promoter and thus induces gene expression. (B) Arabinose inducible gene expression upon active uptake *via* AraE and AraFGH transport proteins. In the presence of arabinose AraC positively regulates P_BAD_ promoter activity, whereas in the absence of arabinose AraC tightly represses target gene expression. (C) Pm/XylS regulated gene expression driven by benzoates that are imported *via* passive diffusion and initiate the XylS regulator-dependent activation of Pm promoter based expression. Abbreviations: *galP*: galactose permease gene; *lacI*: *lac* repressor gene; *lacZYA*: lactose metabolization and uptake genes; *araFGH*: arabinose transporter genes; *araE*: arabinose transporter genes; *araC*: *ara* regulator gene; *araBAD*: arabinose metabolization genes; *xylS*: *xyl* regulator gene; *tolX-H*: toluene degradation operon.

For *E*. *coli*, which is the most commonly applied microbial expression host [5,8], the *lac*-based regulation of expression is typically the first-to-try system for recombinant protein production [9,10]. *E*. *coli* BL21(DE3) [11] and its derivatives [12–14] are the most frequently used strains for high-level protein production that make use of the highly processive T7-RNA polymerase (T7RP) [15]. Usually, the expression of the chromosomally integrated T7RP gene is controlled by the *lac* promoter and the phage polymerase in turn exclusively drives expression of a synthetic T7*lac* promoter, usually present on an additional expression plasmid. Both, *lac* and T7*lac* promoters, are negatively regulated by the LacI repressor, which dissociates from the operator region upon binding of an appropriate inducer [16,17]. Several natural inducers, such as lactose and galactose [18,19], or synthetic inducers such as methyl-1-thio-β-D-galactopyranoside (TMG) [20] and isopropyl β-D-1-thiogalactopyranoside (IPTG) [21] are able to promote gene expression in this system. Uptake of the natural inducers lactose and galactose in *E*. *coli* mainly depends on the lactose (LacY) and galactose (GalP) permeases [22,23]. The synthetic *lac* inducers IPTG and TMG, however, pass the bacterial cell membrane both by diffusion and by LacY-mediated active transport [24] (Fig 1A).

Another widely used expression system in *E*. *coli* is based on the arabinose utilization network, which positively regulates the P_BAD_ promoter controlled gene expression using the AraC regulator protein [7,25]. In contrast to the LacI regulator, which solely represses transcription in the absence of an appropriate inducer, AraC effectively activates and represses transcription, in the presence or absence of arabinose, respectively, thus allowing for extremely fine-adjustable expression levels [7]. The uptake of arabinose mainly occurs by a complex regulated transport system including the AraE and AraFHG transport proteins [26] (Fig 1B). Furthermore, the Pm/XylS system, which originates from the *Pseudomonas putida* TOL meta operon for the degradation of toluenes and benzoates, finds increasing application for controlling gene expression in *E*. *coli* [7,27]. Here, benzoate inducers such as *m*-toluic or salicylic acid [28] bind to the XylS regulator protein that in turn activates Pm-mediated target gene expression. Opposite to previously mentioned *lac* and *ara*-based expression systems, benzoate inducers for the activation of Pm/XylS systems do not depend on active transport systems but enter the cells solely *via* passive diffusion (Fig 1C) [29].

Additional *E*. *coli* expression systems are based on propionate-inducible P_prpB_/PrpR [30,31], rhamnose-inducible P_rhaBAD_/RhaRS [7,32] or tetracycline-inducible P_tetA_/TetR [33] regulatory systems. Due to costly or toxic inducers, a restricted spectrum of expression hosts or the need for coexpression of recombinant transport systems, those systems are less often applied for biotechnological purposes and are thus not subject of this study.

Here, we comparatively analyzed commonly used *E*. *coli* expression systems, namely the P_T7lac_/LacI, P_BAD_/AraC and Pm/XylS systems, in order to gain more detailed knowledge at the single-cell level. We used the synthetic M9CA medium of defined composition to characterize the inducibility of the three expression systems in response to different inducer molecules and investigated the influence of inducer-uptake affecting genetic modifications on phenotypic heterogeneity. Our results provide new insights into individual strengths and weaknesses of each expression system in terms of system responsiveness, growth behavior and phenotypic heterogeneity.

## Materials and Methods

### Microfluidic chip fabrication and experimental setup

Microfluidic polydimethylsiloxane (PDMS) chips incorporating media supply channels of 10 μm height and cultivation chambers of 1 μm height were fabricated by common silicone elastomer molding. Therefore, a 100 mm silicon wafer carrying inverted SU-8 microstructures processed by cleanroom photolithography served as the replication mold. A PDMS base and crosslinker mixture (1:10) was then poured onto the mold and thermally polymerized. After releasing the PDMS slab containing the structure imprint, individual chips were cut and inlet and outlets were punched manually. Before each experiment, PDMS chips were cleaned, oxygen plasma activated and finally permanently bonded to a microscopy cover slide. Detailed information regarding the device layout and fabrication can be found in previous studies [34–36].

Fluidic connections were established by silicone tubing (Tygon S-54-HL, ID = 0.25 mm, OD = 0.76 mm, VWR International) and dispensing needles (dispensing tips, ID = 0.2 mm, OD = 0.42 mm, Nordson EFD). A medium flow rate of approximately 200 nl min^-1^ was generated by a syringe pump (neMESYS, centoni GmbH, Germany). Prior to cultivation, cells at the exponential growth phase (OD_580_ of 0.3–0.5) were inoculated into the chip. Then specific growth chambers which were most suitable for imaging were manually selected, leading to a short delay between the initial induction and start of the experiment. The maximum cultivation duration was determined by the growth rate and the fixed chamber volume.

### Microscopy setup

Microscopy images were taken using an inverted microscope (Nikon TI-Eclipse, Nikon Instruments, Germany) equipped with a 100x oil immersion objective (CFI Plan Apo Lambda DM 100X, NA 1.45, Nikon Instruments, Germany) and a temperature incubator (PeCon GmbH, Germany). Phase contrast and fluorescence time-lapse images were recorded every 10–15 minutes using an ANDOR LUCA R DL604 CCD camera. Fluorescence images were recorded with an exposure time of 200 ms using the Nikon Intensilight as light source with an ND filter of 1/8 (Nikon, Japan) and an appropriate YFP filter (EX 490–550 nm, DM 510 nm, BA 520–560 nm).

### Image and data analysis

Time-lapse movies of monolayer growth chambers were analyzed using a custom, specialized workflow implemented as an ImageJ/Fiji plugin [37]. Cell identification was performed using a segmentation procedure tailored to detect individual rod-shaped cells in crowded populations. Maximum growth rates were derived for each colony by fitting an exponential function to the cell number increase applying the method of least squares [38,39]. Basal expression factors were calculated as ratios of averaged fluorescence values for non-induced expression cultures and non-induced control cultures (lacking the respective expression vector) at the end of the respective experiment. System responsiveness was measured as the positive slope of linear fitting functions for the averaged fluorescence of single-cell fluorescence values increase during the first 60 min of the experiment. The dynamic range of induction was calculated as the highest ratio of averaged fluorescence values for induced and non-induced cultures over the whole course of the experiment.

### Growth Media

Solid Lysogeny Broth (LB) plates were prepared using 25 g l^-1^ready-to-use mix Luria/Miller (Carl Roth, Karlsruhe, Germany) and 15 g l^-1^ agar-agar (Carl Roth, Karlsruhe, Germany).

Liquid cultivations were performed using M9CA medium: 4 g l^-1^ Bacto^™^ casamino acids (BD Biosciences, Franklin Lakes, NJ, USA), 6.8 g l^-1^ Na_2_HPO_4_, 3 g l^-1^ KH_2_PO_4_, 0.5 g l^-1^ NaCl, 1 g l^-1^ NH_4_Cl, adjusted to pH 6.8 at 25°C. Supplementation of 2 mM MgSO_4_ (from separately autoclaved 1 M stock solution) and 8 g l^-1^ glycerol (from sterile-filtrated stock solutions) was performed after autoclaving.

Plasmid-containing strains were maintained by applying 25 μg ml^-1^ of kanamycin in both solid and liquid cultivation media.

### Bacterial strains and plasmids

All bacterial strains, plasmids and oligonucleotides used in this study are listed in S1 Table. The construction of expression vectors and recombinant DNA techniques were carried out in *E*. *coli* DH5α as described by Sambrook *et al*. [40]. To yield a benzoate induction with a broader inducer spectrum and a stronger induction response, an afore-described R45T mutation [28,41] was introduced into the XylS regulator protein *via* overlap extension PCR [42] using Primers 1–4 (S1 Table). The resulting PCR product as well as the target vector pSB-M117-2-g [6] were digested *via Sal*I and *Sac*I restriction. The mutagenized *xylS* PCR product was then inserted into the vector backbone *via* ligation, yielding the vector pM117-R45T-GFP. The resulting construct was verified *via* sequencing. Prior to application of the expression systems listed in Table 1, the corresponding expression vectors were freshly heat-shock transformed into the respective expression hosts.

**Table 1:** *E*. *coli* expression systems characterized in this study.

System	UptakeMechanism	Inducer	Inducer concentrations*	*E*. *coli* strain (plasmid)	Cultivation temperature	References
P_T7lac_/LacI	active*(lacY*^+^*)*	IPTG	0, 0.05, 0.1 mM	BL21(DE3) (pRhotHi-2-EYFP)	37°C	[47]
P_T7lac_/LacI	passive *(lacY*^-^*)*	IPTG	0, 0.05, 0.1 mM	Tuner(DE3) (pRhotHi-2-LacI-EYFP)	37°C	[14]
P_T7lac_/LacI	*active (galP*^+^ *lacY*^+^*)*	galactose	0, 0.4, 1 mM	BL21(DE3)** (pRhotHi-2-LacI-EYFP)	37°C	[14]
P_BAD_/AraC	*active (araEFGH*^+^*)*	arabinose	0, 1, 2.5 mM	Tuner(DE3)*** (pAra-GFPmut3)	37°C	[48]
P_M1-17_/XylS	passive	*m*-toluic acid	0, 0.05, 0.1 mM	Tuner(DE3) (pM-117-R45T-GFPmut3)	30°C	[6,28] & this study
P_M1-17_/XylS	passive	salicylic acid	0, 0.5, 1.5 mM	Tuner(DE3) (pM-117-R45T-GFPmut3)	30°C	[6,28] & this study

* w/o inducer, intermediate inducer concentrations, high inducer concentrations

** *galK*^-^ strain: inability to metabolize galactose, enables sufficient galactose accumulation for induction

*** *araBAD*^*+*^ strain: metabolizes arabinose, increased inducer concentrations are essential

### Precultivation

To obtain comparable microfluidic expression cultures, precultivation was performed exactly as described using fresh LB-Agar transformation plates. First, an overnight preculture was inoculated from a fresh transformation plate in 0.8 ml of the final cultivation medium. After 16 h of cultivation a fresh culture was inoculated in again 0.8 ml of the final cultivation medium to a cell density corresponding to an optical density of 0.01 at a wavelength of 580 nm (OD_580_). This culture was cultivated until an OD_580_ of 0.3–0.5 was reached. Exponentially growing cells were then immediately seeded into the microfluidic cultivation chips. All precultivations (30 or 37°C, 1500 rpm) were performed in sterile 48-well flowerplates (m2p-labs GmbH, Aachen, Germany) using a deep-well plate incubator (Thermomixer C; Eppendorf, Hamburg, Germany).

## Results

In synthetic biology and biotechnology, expression processes are mainly observed on average-based population scale, thus ignoring phenotypic heterogeneity especially in case of adequate overall yields and functionality. However, cell-to-cell heterogeneity may distinctly hamper overall product yields [43]. This becomes most evident for toxic gene products [44] and if non-producing cells overgrow the culture due to a faster growth [45]. Unraveling phenotypic heterogeneity is therefore a key aspect in understanding and optimizing recombinant protein production.

In order to precisely analyze expression systems at single-cell level, cells have to be characterized under well-defined environmental conditions, enabling one to distinguish between phenotypic (intrinsic) and environmental (extrinsic) heterogeneity [36,43].

**Fig 2:**
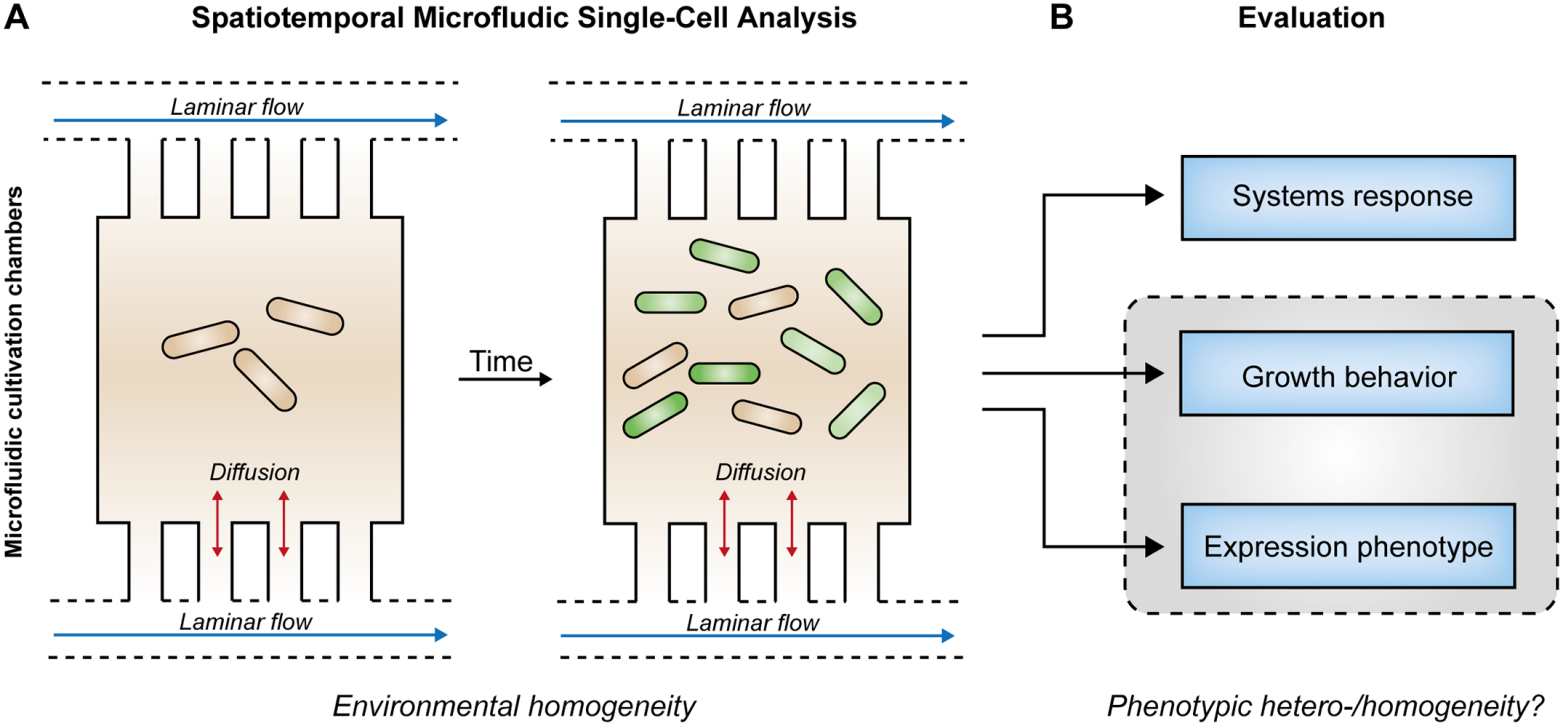
Microfluidic single-cell cultivation experiments. **A)** Spatiotemporal microfluidic single-cell analysis of isogenic populations enables well-defined environmental conditions (environmental homogeneity) within growth chambers due to constant laminar media flow through nutrient supply channels. **B)** Exact evaluation of expression systems response, growth behavior and expression phenotype to expose phenotypic heterogeneity (grey box) of analyzed expression systems.

This challenge was strikingly tackled in recent years by means of microfluidic single-cell cultivation approaches. These allow for cultivations under precisely controlled cultivation conditions implemented by continuously perfused cultivation medium (Fig 2A). Here, laminar flow conditions and diffusion-dominated mass transport lead to well predictable environmental homogeneity. Furthermore, microfluidics in combination with time-lapse imaging facilitates the analysis of cellular behavior and physiology with high spatiotemporal resolution [34–36]. We thus employed novel microfluidic bioreactor systems [36] for cultivation and *in vivo* fluorescence reporter-based monitoring of gene expression in common *E*. *coli* expression systems to uncover system-inherent specifications including responsiveness, growth behavior and expression phenotype (Fig 2B).

Complex growth media such as LB medium are widely used for the cultivation of *E*. *coli* in both bulk and single-cell analysis. In contrast to synthetic media, they contain yeast extracts, which are chemically not accurately defined, thus limiting exact knowledge about the nutrient composition. Moreover, distinct variations between different yeast extract suppliers or lots might occur [46]. Our preliminary analyses of the carbohydrate composition of different LB cultivation media (S2 Table, S1 Appendix) and its impact on expression strength and homogeneity (S1 Fig) revealed striking differences.

These results clearly demonstrate that complex LB cultivation media should not be applied for microfluidic cultivations where precise control over gene expression is of primary interest; we therefore selected for growth of *E*. *coli* the synthetic M9CA as an alternative medium, which lacks in residual carbohydrates (S2 Table). M9CA is a rich cultivation medium that contains well-defined components that can be individually adjusted if necessary and enables fast growth of *E*. *coli* cells.

### Comparative system specification analysis of selected *E*. *coli* expression systems

With microfluidic single-cell analysis and the synthetic M9CA medium we choose a well-defined experimental setup, providing high environmental homogeneity, to enable detailed insights into relevant microbial expression systems on the single-cell level.

Hence, we comparatively analyzed system-inherent specifications of a defined set of commonly applied *E*. *coli* expression systems using different inducer molecules, concentrations and uptake mechanisms (Table 1). In contrast to other studies focusing on high-transformation efficiency, low background of target gene expression [6] or natural P_*lac*_ constructs with *E*. *coli* K12 wildtype strain derivatives [21], we solely analyzed expression systems that were based on the most commonly used high-level production host in biotechnology, namely *E*. *coli* BL21(DE3) and its *lacZY*^-^ derivative Tuner(DE3). These two strains, in contrast to commonly used K12 strains, are deficient in the proteases encoded by *ompT* and *lon*, which has proven beneficial for high-level protein production [9]. Moreover, due to the implementation of the highly processive T7RP, the strains are well suited for applying the frequently used expression vectors harboring hybrid T7*lac* promoters for target gene expression (P_T7lac_/LacI system).

P_T7lac_/LacI-based IPTG induction of target gene expression was analyzed in both BL21(DE3) (*lacY*^+^) and Tuner(DE3) (*lacY*^-^) expression strains since previous studies indicated crucial differences in responsiveness and phenotypic heterogeneity [14]. Whereas the *lacY*^+^ system represents the ‘what to try first’ *E*. *coli* expression system, the here applied *lacY*^-^ system was expected to bear improved expression features due to the absence of permease LacY and elevated amounts of repressor LacI. We further analyzed galactose induction in the *lacY*^+^ system as well as an arabinose inducible P_BAD_/AraC system [48]. Moreover, we tested benzoate induction using a Pm/XylS system with the high-level expression promoter P_M117_ [6,27]. To enable a promiscuous benzoate induction with diverse benzoate derivatives [28], in particular to empower salicylic acid induction in addition to conventional *m*-toluic acid induction, we introduced an R45T mutation into the XylS regulator protein. For the non *lac*-based expression systems, we consistently used the *lacY*^-^ *E*. *coli* strain Tuner(DE3) as it exhibits strict inhibition of (in this case) undesired T7RP gene expression under the here applied conditions.

To uncover system-inherent specifications for all analyzed *E*. *coli* expression systems, microfluidic cultivations were compared using no inducer as well as intermediate and high inducer concentrations (for exact concentrations and setups see Table 1). All cultivations were conducted at 37°C, except for benzoate induction systems which worked best at 30°C.

First, we aimed to analyze the system responsiveness of the respective expression systems since temporally precise control is of utmost importance for several synthetic biology and biotechnological applications. For instance, in rapidly growing cultures, exclusively prompt induction responses might enable sufficient product formation prior to nutrient depletion or the transition into the less productive stationary phase.

**Fig 3:**
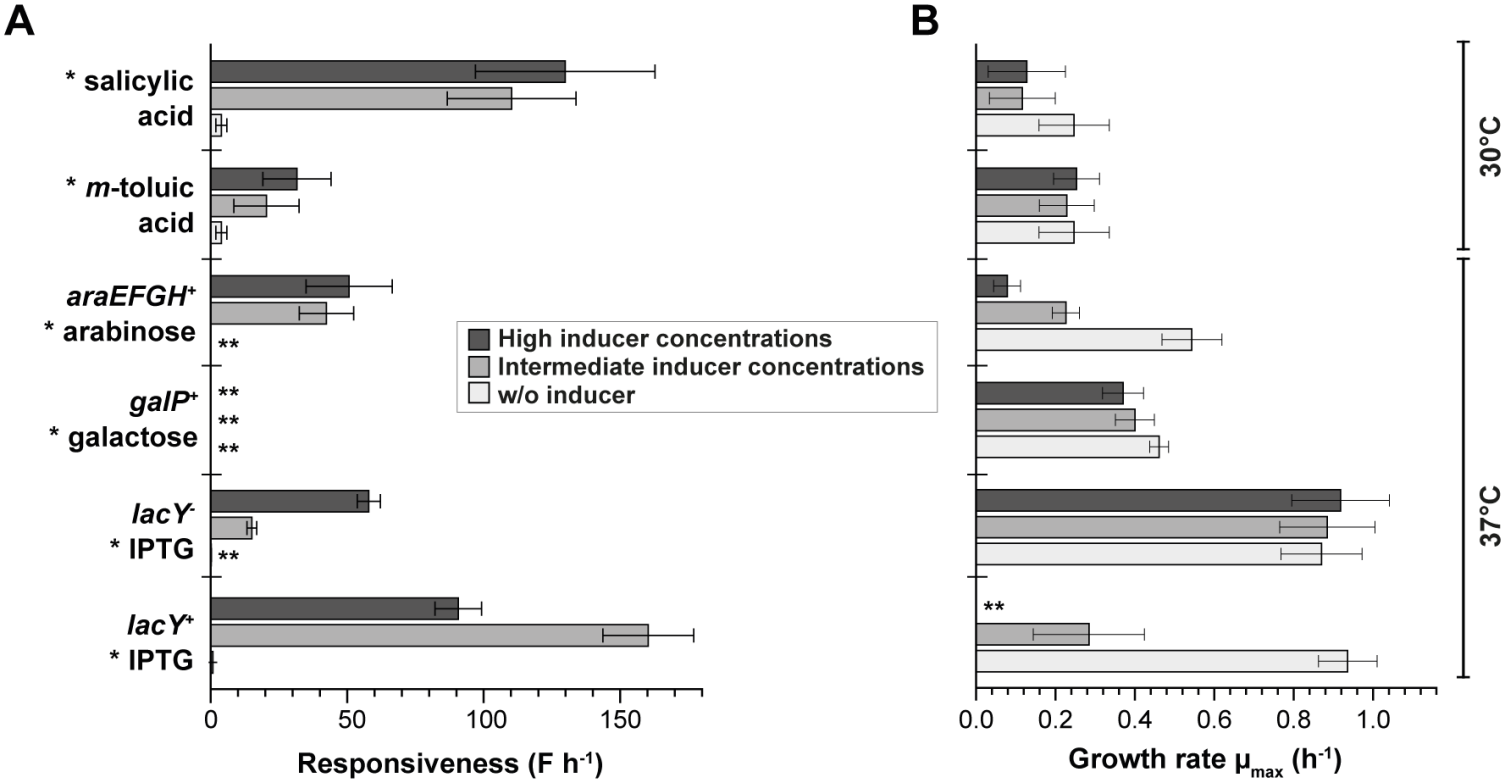
System responsiveness and growth analysis of characterized *E*. *coli* expression systems. **A)** Responsiveness was calculated using the initial linear slope of the averaged single-cell fluorescence increase in the first 60 min of cultivation. **B)** For the correlation between cellular growth and the level of induction, growth rates were calculated for at least 10 populations of microfluidic expression cultures without inducer (light grey), as well as with intermediate (grey) and high inducer concentrations (dark grey). Mean and standard deviations derive from 10 individual colonies. Inductors are labeled by asterisks (*). Double asterisks (**) indicate that no calculation was possible.

Thus, the system responsiveness (Fig 3A) was evaluated using the initial increase of single-cell fluorescence (linear slope of fluorescence for the first 60 min) for all six expression strains (Table 2). IPTG induction of the P_T7lac_/LacI system using the *lacY*^+^ strain *E*. *coli* BL21(DE3) showed the strongest initial target gene expression response. Notably, a likewise rapid and strong response was observed for salicylic acid induction of the Pm promoter, albeit the here applied lower cultivation temperature. An intermediate responsiveness of gene expression was observed for IPTG induction with the *lacY*^-^ system, as well as for arabinose induction and *m*-toluic acid induction. For galactose induction no detectable increase of fluorescence was initially monitored, and only a slight increase occurred over the course of cultivation (S2 Fig).

**Table 2:** System responsiveness, growth interference, basal expression and dynamic range of different *E*. *coli* expression systems. Values were calculated using fluorescence values obtained during microfluidic cultivation of at least 10 microcolonies. All shown data were obtained from highest values (see Fig 3 and S2 Fig for details and respective maxima).

System	Systems responsiveness [F h^-1^]	Growth interference** [x-fold reduction]	Basal expression factor	Dynamic range
LacY^+^ & IPTG	160.3	>> 3.3 *	2.1	63.4
LacY^-^ & IPTG	57.9	n.d.	1.4	67.0***
galactose	2.3	1.2	1.3	2.3
arabinose	50.7	7.0	1.0	106.6
*m*-toluic acid	31.6	1.1	8.9	5.4****
salicylic acid	129.9	2.1	8.9	27.2****

* difficult to determine due to complete growth arrest

** calculated from Fig 3B by comparing cultivations without inducer and with high inducer concentrations

*** due to fast growth and thus short cultivation times, expected to significantly increase in long-term setups [14]

**** might be improved by the application of the low background wildtype Pm promoter [6]

In addition to the velocity of induction response, the interplay between growth and target gene expression is a key aspect that decisively affects the productivity of a given bioprocess. Here, slowly growing overproducers might fall behind with respect to overall yields due to poor biomass formation. It is thus essential, that production of target proteins does not result in substantial inhibition of cellular growth.

Hence, we subsequently evaluated growth of respective expression cultures (Fig 3B) and compared it without inducer as well as using intermediate and high inducer concentrations (see Table 1). Strikingly, tremendous growth interferences were revealed for BL21(DE3) (*lacY*^+^) when cultures were supplemented with IPTG, which seemed to correlate with the strength of induction since intermediate inducer concentrations already resulted in a 3.3-fold reduction of growth whereas high inducer concentrations almost completely abrogated growth (Table 2). A similarly strong growth impairment was observed for arabinose induction of target gene expression as intermediate and high inducer concentrations decreased growth 2.4- and 7.0-fold, respectively. In contrast, minor effects on cellular growth were observed for galactose (up to 1.2-fold reduction) and salicylic acid induction (up to 2.1-fold reduction), respectively. Remarkably, induction with *m*-toluic acid (up to 1.1 fold reduction) and especially IPTG induction using the *lacY*^-^ system revealed hardly any interference with growth compared to respective non-inducing conditions (Table 2). Further, growth rates were generally about 3.8-fold decreased for reduced working temperatures of 30°C (μ_max_ = 0.25 ± 0.09 h^-1^) as compared to cultivations at 37°C (μ_max_ = 0.94 ± 0.07 h^-1^) (Fig 3B).

Conclusively, analysis of systems responsiveness and cellular growth during microfluidic cultivation revealed that the most rapidly responding expression systems, namely the P_7lac_/LacI system using the *lacY*^+^ strain BL21(DE3) as well as the salicylic acid induction system, suffer from a significant growth impairment upon induction. In some production processes this could lead to low overall yields due to poor development of biomass. In this context, moderately responding expression systems, such as the *m*-toluic acid induction system or especially IPTG induction using the *lacY*^-^ system, may prove beneficial with respect to overall productivity.

In general, the interplay between growth and protein production might even be enlarged for toxic proteins, so that a low background expression activity is highly favorable. In this context, a full inhibition of basal target gene expression in the absence of specific inducers, allowing sufficient biomass formation prior to induction of the protein production process, is an important prerequisite for a robust bacterial expression system.

We thus further calculated basal expression factors as fluorescence ratios of strains harboring respective expression plasmids under non-inducing conditions and corresponding strains without expression plasmid. IPTG induction using the *lacY*^+^ system was moderately leaky (2.1-fold), whereas IPTG induction with the *lacY*^-^ system (1.4-fold) as well as galactose induction (1.3-fold) showed a low basal expression. Noteworthy, for those two later systems, a modified expression vector providing elevated amounts of the LacI regulator [14] was applied. In contrast, the here selected promiscuous (XylS R45T) benzoate induction system using the high-level P_M117_ promoter [27] revealed a significantly leaky expression with basal expression factors of up to 8.9 (Table 2). Notably, the wildtype Pm promoter instead of the here applied mutagenized high-level expression variant should offer a reduced leakiness [6]. The tightest promoter observed during microfluidic cultivations was the arabinose inducible P_BAD_ as no basal expression could be detected.

To further evaluate the controllability of expression response, the dynamic range of the expression response was quantified as the ratio of the maximum fluorescence (upon induction) and the basal fluorescence of non-induced cultures. The dynamic range of induction was highest for arabinose induction (up to 107), and remarkable for both IPTG induction in the *lacY*^+^ system (up to 63) and the *lacY*^-^ system (up to 67). A moderate dynamic range of gene expression response, was observed for salicylic acid induction (up to 27), whereas *m*-toluic acid (up to five) and especially galactose induction (up to two) showed poor inducibility. The moderate dynamic ranges for the generally (in absolute fluorescence values) well inducible benzoate induction systems, in terms of both expression strength and responsiveness (S2 Fig), mainly emerge from the high basal expression levels. In contrast, galactose induction is slow and extremely weak under applied conditions. For IPTG induction using the *lacY*^-^ system it should be further noted that the rapidly responding system exhibits fast growth so that the respective values were calculated after cultivation times of only 3 h. In general, the dynamic range is expected to rise with the course of cultivation in microfluidic setups for long-term cultivations [49,50].

### Uncovering expression heterogeneity in selected *E*. *coli* expression systems

**Fig 4:**
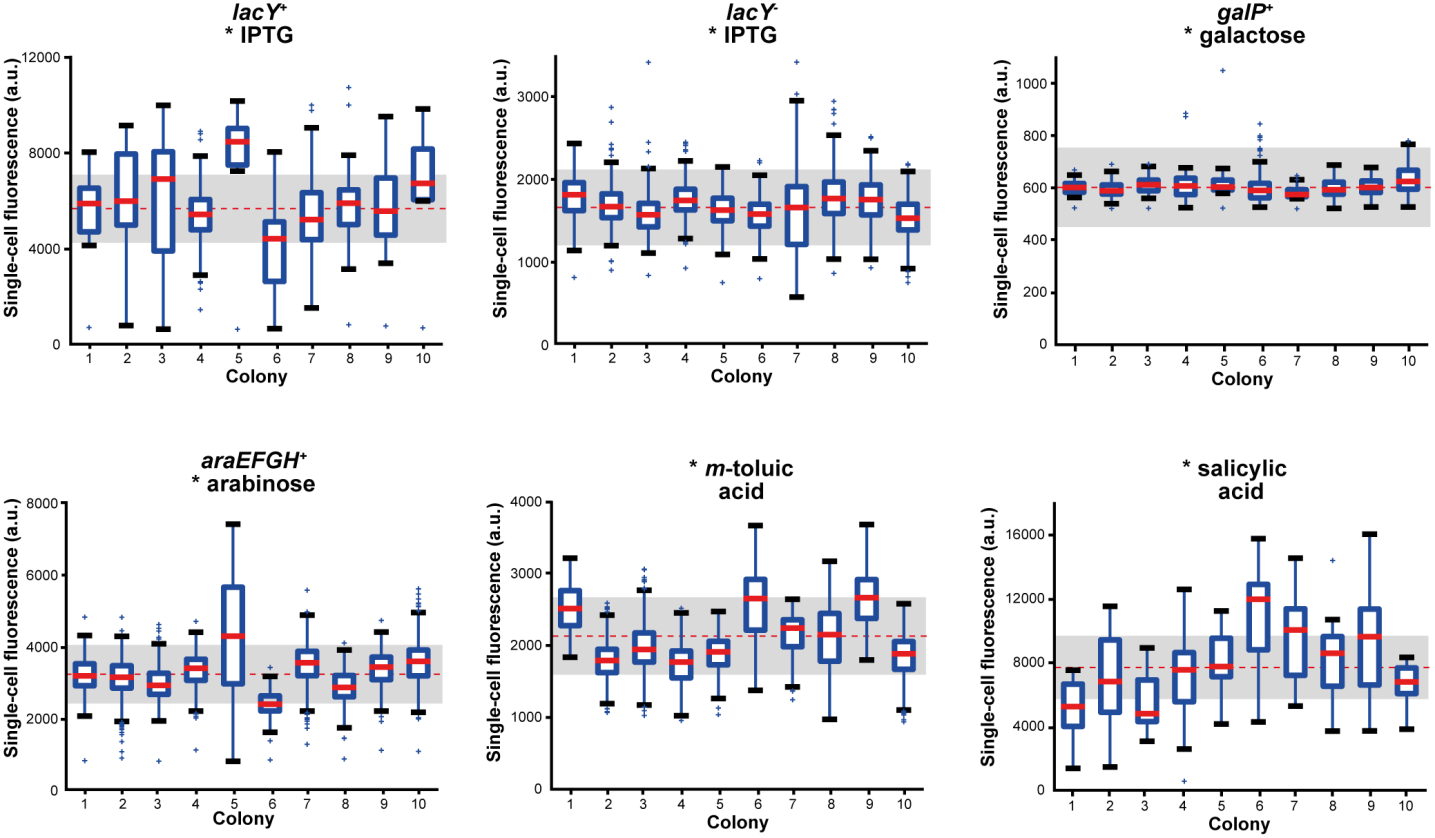
Box plot analysis depicting cell-to-cell variations in gene expression for different optimally induced *E*. *coli* expression systems. Cell-to-cell fluorescence distributions of optimally induced expression systems are depicted with the total mean (dotted red line) and the spread interval (25% of mean, grey box) for ten individual microcolonies evaluated at the end of each experiment (end point criteria: cultivation chambers fully filled with cells or μ_max_ ~ 0). Exact inducer concentrations for optimal induction were 0.1 mM IPTG (for each system), 1 mM galactose, 1 mM arabinose, 0.1 mM m-toluic acid and 1.5 mM salicylic acid. For each individual colony, medians (bold red line) indicate values above which 50% of cells are located, blue boxes indicate interval into which 50% of fluorescence values fall. Top or bottom of the box show areas, where 25% of cells are located above or below, respectively. Antenna indicate the 1.5-fold interquartile distance (IQR, 1 IQR = box height) or the last data point detected inside the 1.5-fold IQR. Outliers outside of the 1.5-fold IQR were marked as crosses.

Upon characterization of valuable expression system specification parameters such as responsiveness, effect of protein production on growth, promoter tightness and dynamic range of induction in a bulk single-cell analysis, the focus was subsequently laid on cell-to-cell variations during the expression response within a specific *E*. *coli* microcolony. To this end, the single-cell fluorescence distributions were comparatively analyzed in ten individual microcolonies for all six expression systems, respectively. Due to a slight fluorescence reduction for high inducer concentrations (S2 Fig), which was likewise observed in literature [26,51], the P_BAD_/AraC system was analyzed for intermediate inducer concentrations, whereas all other expression systems were analyzed for high inducer concentrations. The results of the fluorescence distribution analyses are shown as a boxplot for a descriptive depiction of the recorded data sets (Fig 4). IPTG induction in the *lacY*^+^ system led to a high number of cells that significantly deviated from the mean fluorescence (red dotted line) and the coefficient of variation (CV) interval of 25% (grey box), where only 63% of all data fitted in.

For IPTG induction in the *lacY*^-^ system 83% of all single-cell fluorescence values fell into the 25% CV interval, beyond which merely individual outliers were detected. The fit into the 25% CV interval was even more distinct for galactose induction (98%), yet an overall poor inducibility was detected and a rather separate evaluation might be appropriate. Arabinose induction revealed a moderate fluorescence distribution as the majority of cells exhibited average fluorescence levels (77%). Some colonies, however, significantly deviated from the mean and showed a strikingly increased deviation.

The same is true for *m*-toluic acid induction *via* the Pm/XylS system as medians generally varied inside of the 25% CV interval (79%). For the same Pm/XylS system, salicylic acid induction revealed a much more wide-spread single-cell fluorescence distribution (just 42% lay within the interval). Half of all medians did not fit into the CV interval and antenna indicated distinct variations.

Boxplot diagrams therefore proved as a suitable depiction to describe cell-to-cell differences in the expression response of single-cell cultivations from different *E*. *coli* expression systems upon induction. Evidently, heterogeneous expression systems showed a significant quantity of cells outside the selected 25% CV interval (grey box).

**Fig 5:**
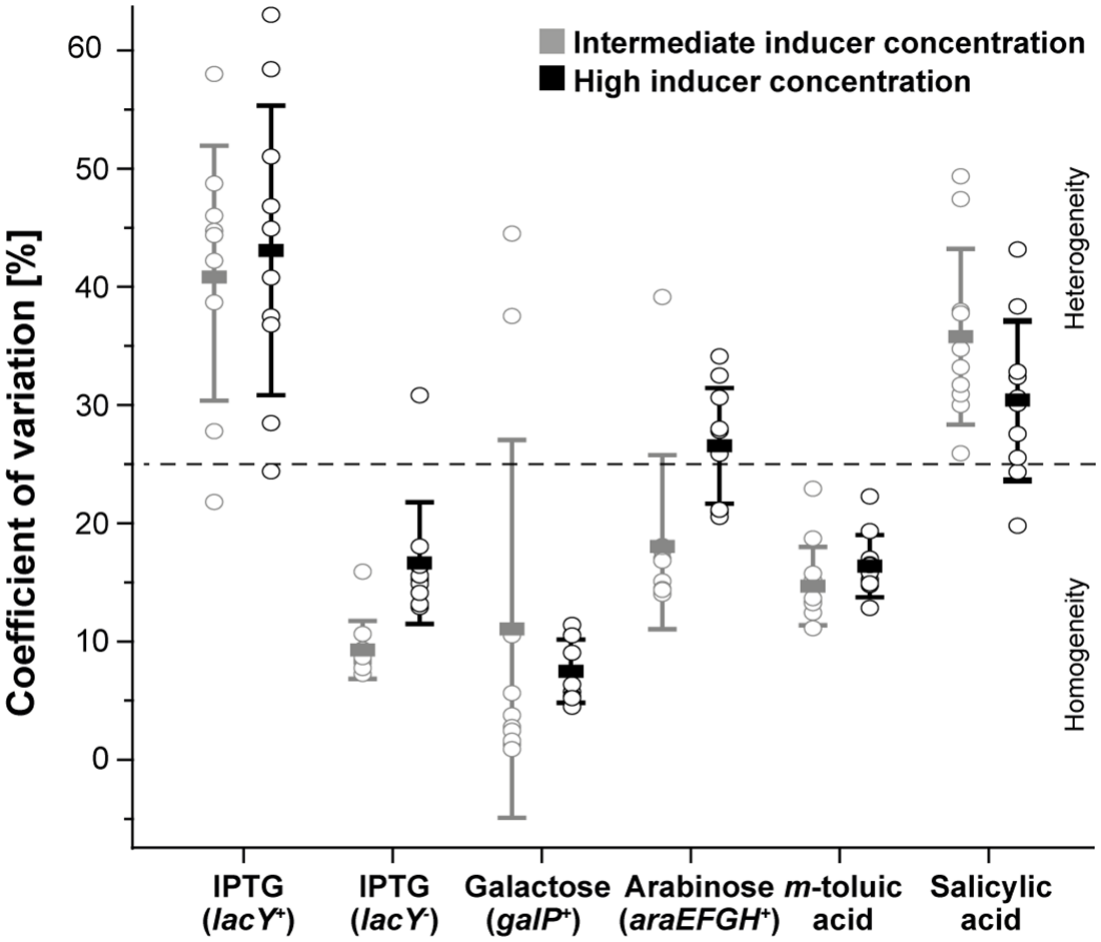
Expression heterogeneity analysis of different *E*. *coli* expression systems during microfluidic cultivation for intermediate (grey) and high inducer concentrations (black). CVs for ten individual colonies (open circles) are plotted together with the respective overall mean (bold dash) and the corresponding standard deviation. The grey dotted line indicates the threshold for expression heterogeneity (CV > 25%) above which colonies are considered as heterogeneous.

In a next step, we intended to further classify and rank the expression systems with respect to expression heterogeneity. We thus aimed to identify further quantitative parameters suitable for a conclusive determination of expression homogeneity or heterogeneity, respectively. To this end, we determined the normed coefficient of variation (CV) as well as the number of outliers as significant parameters to visualize and appropriately identify system-inherent cell-to-cell variations. First, the CV was used to roughly assign homogeneity or heterogeneity to the respective expression system. For intermediate inducer concentrations (Fig 5), the *lacY*^-^ system with IPTG induction revealed the smallest CV observed (9 ± 2%), indicating homogeneity. Similarly low CVs were found for *m*-toluic acid (14 ± 3%) as well as galactose (11 ± 16%) and arabinose induction (18 ± 7%). Significantly higher CVs, and thus a rather heterogeneous expression behavior, were observed for salicylic acid (36 ± 8%) and IPTG induction with the *lacY*^+^ system (41 ± 11%).

For high inducer concentrations (Fig 5), galactose induction showed the lowest CV (7 ± 3%). Due to poor inducibility, however, it is difficult to evaluate the expression heterogeneity appropriately. In contrast, *m*-toluic acid (16 ± 3%) and IPTG induction with the *lacY*^-^ system (17 ± 5%) showed low CVs together with appropriate inducibility so that their expression responses can be characterized as clearly homogeneous. A rather heterogeneous expression response was observed for arabinose (26 ± 5%) and salicylic acid induction (30 ± 7%), whereas a distinct expression heterogeneity was depicted for IPTG induction using the *lacY*^+^ system (43 ± 12%).

Outliers exhibited a rather chaotic distribution in the plots and did not follow the expectation that homogeneity would go along with a low number of outliers and heterogeneity in reverse, with a high number of outliers (S5 Fig). Most evident examples were galactose induction, which depicts an increased number of outliers despite a very low CV, or salicylic acid induction, which just sporadically showed outliers despite obvious expression heterogeneity. As no direct correlation between outliers and the CV could be obtained and outliers seemed further specific for some expression systems, the fraction of outliers proved rather unsuited as a criterion for the evaluation of expression heterogeneity. It rather seems that the number of outliers correlated with system-specific rare heterogeneity events such as low inducibility (e.g. for galactose induction) or cellular stress due to high expression levels (e.g. for high inducer concentrations with arabinose and IPTG in the *lacY*^+^ system).

In this context, however, IPTG induction using the *lacY*^-^ system as well as *m*-toluic acid induction showed most robust expression performances as they constantly exhibited low CVs and negligible fractions of outliers (bottom left quadrants in S5 Fig) irrespective of the applied inducer concentration. Moreover, for IPTG induction using the *lacY*^+^ system and for arabinose induction it became evident that the degree of induction influenced the fraction of outliers, as higher inducer concentrations led to increased numbers of outliers (S5 Fig).

**Fig 6:**
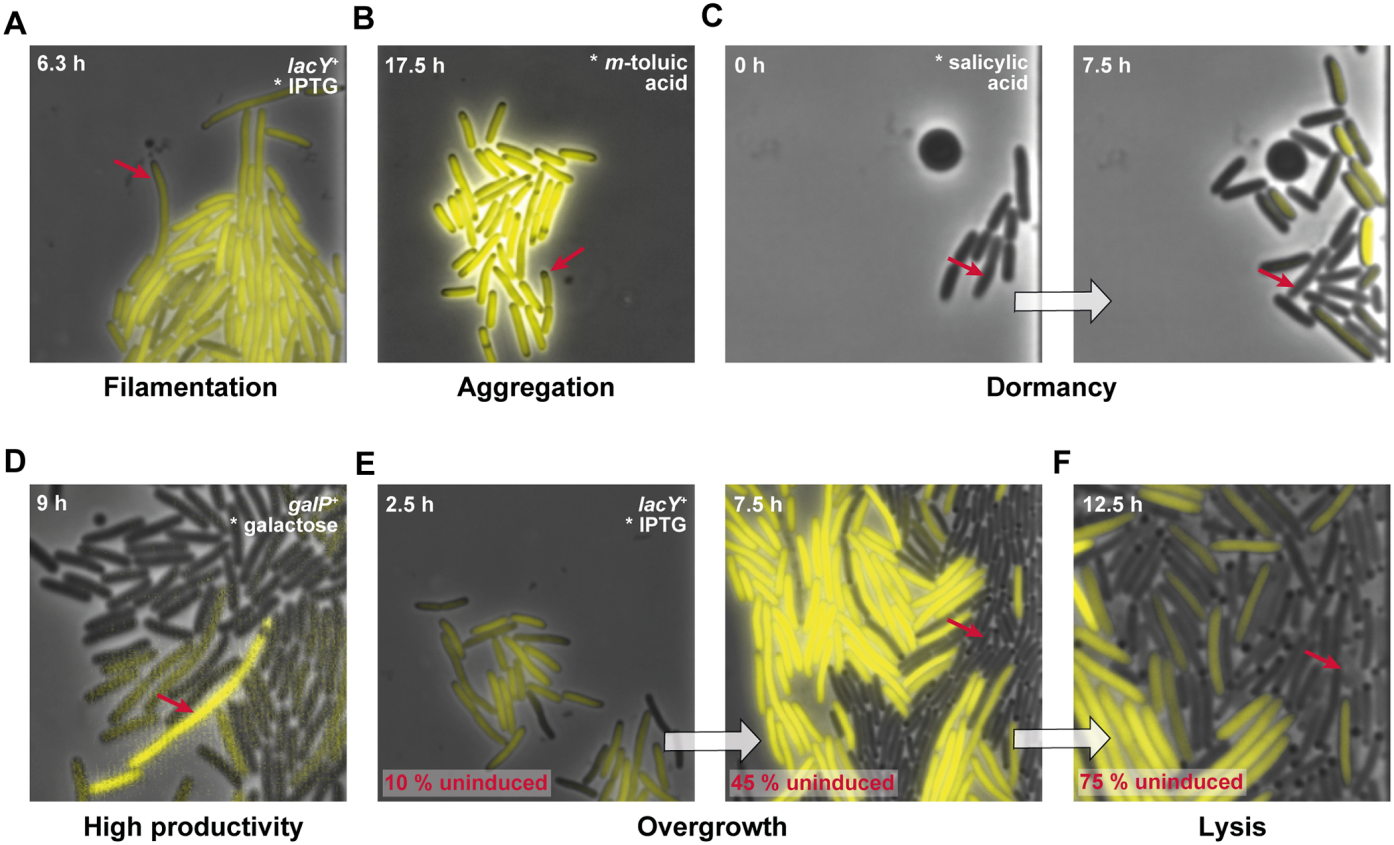
Rare cell-to-cell variation phenomena selected from conducted microfluidic analyses. (A) Filamentous cells that grow but do not divide. (B) Formation of dark spots indicating aggregates in highly producing cells. (C) Dormant cells, which are significantly delayed or irresponsive in growth and expression. (D) Highly producing cells in an otherwise sparely producing population. (E) Overgrowth of slowly—dividing producer cells by rapidly growing non-producers. (F) Cell lysis of stressed overproducer cells or even rapidly growing non-producer cells. Red arrows indicate cells exhibiting the respective phenomena.

Taking into account both visual and statistical analyses of expression heterogeneity, the selected CV (22 ± 5% for all systems on average) threshold of 25% (roughly average plus deviation) seemed appropriate for the characterization of expression homogeneity. For the fraction of outliers (3.4 ± 2.9% on average) more than 6% (roughly average plus deviation) appeared unusual for both homogeneous and heterogeneous expression systems and may be seen as an indicator of lacking systems robustness and of rare cellular events such as spontaneous mutations or rare phenotypes. Therefore, rare phenotypes observed during here conducted microfluidic cultivations were subsequently compiled to provide insights into unusual phenomena during employment of an inducible expression system (Fig 6). Rare heterogeneities of cell phenotypes that were observed during microfluidic cultivations include cell filamentation (Fig 6A), protein aggregation (dark non-fluorescing spots), which is potentially attributed to inclusion body formation (Fig 6B), dormant cells that rest in growth and expression (Fig 6C), single cells that show a high productivity within sparely producing cells (Fig 6D), or sudden cell lysis (Fig 6F).

The phenomenon of overgrowth (Fig 6E) clearly illustrates why phenotypically homogeneous expression systems are crucial for the optimization of synthetic and systems biology as well as biotechnological applications. For growth-interfering overexpression, it becomes apparent that rapidly dividing non-producers can outperform the number of slowly growing producers during the course of cultivation, distinctly reducing overall product yields. This important observation became evident only by applying microfluidic single-cell analysis with its high spatiotemporal resolution. Specifications of all tested *E*. *coli* expressions systems during microfluidic cultivation are summarized in Table 3.

**Table 3:** Summary of system specifications of *E*. *coli* expression systems during microfluidic cultivation.

Inducer (System)	Responsiveness	Strength	Tightness	Working concentration	Growth impairment	Population
IPTG (*lacY*^+^)	**+++**	**+++**	++*	low	very high	heterogeneous
IPTG (*lacY*^-^)	**++**	**++**	+++*	low	very low	homogeneous
Galactose (*galP*^+^)	**-**	**-**	**++**	high	moderate	n.d.***
Arabinose (*araEFGH*^+^)	**++**	**++**	++*	high	high	partly homogeneous
*m*-toluic acid	**+**	**++**	-**	moderate	very low	homogeneous
Salicylic acid	**+++**	**+++**	-**	high	high	heterogeneous

* If leakiness has to be reduced further, glucose supplementation can be applied [25].

** The wildtype P_M_ Promoter (instead of the P_M117_) can be applied for reduced basal expression [6].

*** Due to poor inducibility during microfluidic cultivation exact evaluation is impeded.

## Discussion

Unraveling phenotypic heterogeneity is a key aspect for the optimization of biotechnological and synthetic biology applications; however, well-defined conditions have to be applied to avoid the influence of environmental heterogeneity on microbial expression setups.

In this study, we demonstrated that cultivation in the synthetic M9CA medium and spatiotemporal microfluidic single-cell analysis provide a constant and homogeneous environment allowing for an extensive comparative analysis of *E*. *coli* expression systems at the single-cell level. We could identify distinct differences in performance relevant parameters of diverse systems and have uncovered distinct differences in responsiveness, controllability and homogeneity of target gene expression (Table 3). Interestingly, the most commonly applied P_T7lac_/LacI expression system based on *E*. *coli* BL21(DE3) clearly exhibited significant deficits with respect to expression homogeneity and growth. Throughout the whole cultivation, significant cell-to-cell variations of target gene expression were observed for both intermediate and high inducer concentrations. A similar system based on the lactose permease LacY-deficient *E*. *coli* strain Tuner(DE3), however, showed a remarkable homogeneity with regard to both expression and growth. Here, the beneficial features of this strain could be clearly attributed to the absence of LacY as the *lacY*^+^ system using likewise elevated amounts of LacI depicted similar expression heterogeneity as the original *lacY*^+^ system (S6 Fig).

In addition, promoter tightness under non-inducing conditions as well as robustness of cellular growth during protein production of this system distinctly outperformed all other monitored expression systems. A favorable performance was also observed for the tested benzoate inducible Pm/XylS system, as *m*-toluic acid induction produced a clearly homogeneous, rapid and strong expression response. The choice of benzoate inducer, however, was crucial for the systems performance as the alternative benzoate inducer salicylic acid evoked an even stronger but also highly heterogeneous target gene expression, which resulted in distinctly impaired cellular growth. Arabinose induction *via* the P_BAD_/AraC system, in turn, yielded a strong and only partly homogeneous expression response. Growth impairment for high inducer concentrations was relatively high, though. The galactose-inducible *E*. *coli* expression system was found not to be suited for microfluidic perfusion but well-functioning in batch cultivations (S3 Fig). Thus, inducer uptake might be impeded by the continuous perfusion of inducer supplemented cultivation medium or inducibility might be reduced, in general, by the cells being basically trapped in the exponential growth state, which might for instance interfere with galactose uptake. Here, microfluidic batch cultivations might be an opportunity to unravel system inherent differences with regard to the respective cultivation mode in further detail [52]. To the best of our knowledge, this is the first description of galactose and salicylic acid based induction systems analyzed by microfluidic single-cell cultivation. It has to be noted that systems performances may differ for induction in other media and, in particular, in discontinuous cultivation approaches. This becomes most evident for galactose induction as microfluidically grown cells in the synthetic M9CA medium revealed only poor induction, whereas conventional batch cultivation produced a significant expression response (S3 Fig).

Compared to existing studies using other cultivation media, such as LB [6,14] or minimal medium [21,53], and different single-cell analysis tools, we detected comparable features for our *lac*-based expression setups. Flow cytometric analysis of *lac* expression systems with *lacY*^+^ [6] and *lacY*^-^ [21] strains as well as microfluidic cultivations [14] ascribe similar expression characteristics to both variants, with and without the LacY transporter, for IPTG or TMG induction. Interestingly, the overexpression of *lacY* also appears to be a valuable alternative to gene deletion for implementation of homogeneous expression with *lac*-based gene expression circuits [54]. In contrast to the here depicted results, arabinose induction is mostly described in literature as being heterogeneous [6,48,55]. Here, we found partly homogeneous arabinose induction for the tested arabinose-metabolizing strain *E*. *coli* Tuner(DE3). Presumably, this homogeneous response is due to the presence of the *araBAD* genes encoding the arabinose metabolizing operon, and the choice of the specific expression host strain resulting in increased arabinose concentrations. For lower arabinose concentrations in *araBAD*-deficient strains, expression is known to be heterogeneous and thus extensive work has been invested to achieve a homogeneous arabinose-induced gene expression response by means of AraE transporter overproduction [55,56], mutagenized LacY transporter variants [57] or novel photocaged arabinose inducers [48]. As complex inducer uptake systems have repeatedly been shown to cause expression heterogeneity [14,55,56], easily membrane-permeable photocaged inducers, that bypass specific uptake systems, enable a more homogeneous expression response [14,48]. As another advantage of photocaged inducers, induction processes might be simplified in handling due to the non-invasive and straightforward applicability of light exposure. Especially, where experimental evaluation of diverse, e.g. temporally variable, induction setups is required, rapid triggering of hundreds of different cultures grown in parallel typically causes labour-intensive effort with conventional inducers. In the future, novel optogenetic methods offer to remedy these efforts, and moreover enable attractive control over single cells with high spatiotemporal resolution [14,48].

Microfluidic single-cell analysis proved to be a powerful tool to unravel limitations of biotechnological production processes on single cell level before [43,45]. This study further corroborates that microfluidics methodology is of utmost importance to fully optimize control over bacterial response circuits for biotechnological production processes or synthetic biology applications. Besides the determination of valuable system-inherent specifications for different *E*. *coli* expression systems based on single-cell data, the technique enabled us to zoom in to cell-to-cell variations and their development over time, and finally allowed uncovering rare cellular phenotypes.

Gained in-depth insights will inevitably encompass the optimization of recombinant protein production approaches in the future. Here, phenotypically homogeneous expression systems such as the modified *lacY*-deficient P_T7lac_/LacI as well as the Pm/XylS system with conventional *m*-toluic acid induction might emerge as key players for precise and robust triggering of bacterial gene expression in *E*. *coli* in a homogeneous fashion.

## Supporting Information

S1 Appendix Supporting methods. Exact LB growth media recipes and quantification of galactose, lactose and glucose.
(PDF)

S1 Fig Expression responses and growth of *E*. *coli* BL21(DE3) with (A-C) and without (D) the pRhotHi-2-EYFP expression vector in different complex LB cultivation. (A) Representative micro-colonies, weakly induced (2.5 μM) with IPTG after approximately 4 h of cultivation in four different LB media. (B) Mean fluorescence distribution for the representative microcolonies shown above. Mean values and coefficient of variations are plotted above the bar, indicating the complete spread. (C) Mean fluorescence for ten EYFP-expressing colonies cultivated in the four different media. (D) Comparison of maximum growth rates for non-induced cultivations in the different LB media (grey bars) with growth rates obtained for uninduced cultivation in the novel defined rich medium M9CA (dark grey bars).
(TIF)

S2 Fig Fluorescence profiles for conducted microfluidic expression setups. Averaged single-cell fluorescence development for at least ten populations cultivated without (blue), as well as using intermediate (green) and high inducer concentrations. Shaded areas indicate respective standard deviations. The end of the experiment corresponds to the time were cultivation chambers are almost fully loaded or where cells completely stopped growing.
(TIF)

S3 Fig Bulk fluorescence profiles for batch cultivations of different *E*. *coli* expression systems. Expression response of the selected expression systems 1–6 (**A-F**) in a BioLector microbioreactor system (m2plabs, Germany) under constant monitoring of biomass accumulation and reporter fluorescence. Indicated fluorescence was biomass-normalized. Expression cultures were inoculated to cell densities corresponding to an optical density of 0.05 at 580 nm. Gene expression was induced when cell cultures reached the logarithmic growth phase (cell density of OD580 ~0.5). Cultures induced with 1 mM arabinose start to consume arabinose, while the are still growing, whereas induction with 2.5 mM arabinose leads to tremendous growth impairment and thus no arabinose consumption was observed during the observation period of 10 h. Expression cultures were performed at least in triplicates. Shaded areas indicate respective standard deviations. a.u.: arbitrary units.
(TIF)

S4 Fig Time-resolved fluorescence reporter expression patterns of microfluidic cultivations using intermediate and high inducer concentrations. Histograms were plotted using single-cell fluorescence values obtained from representative populations at the initial (blue, N>8), intermediary (green, halftime of experiment) and end state (red, μ_max_ ~ 0) of conducted microfluidic cultivation experiments.
(TIF)

S5 Fig Expression heterogeneity analysis of different *E*. *coli* expression systems during microfluidic cultivation using (A) intermediate and (B) high inducer concentrations for induction of target gene expression. Percentaged coefficient of variation and fraction of outliers (outside the 1.5-fold IQR) are plotted as potential indicators of expression heterogeneity for ten individual microcolonies. Cross lines reveal respective means and standard deviations. Grey dotted lines show thresholds for expression heterogeneity (CV > 25%) or increased number of rare events (outliers > 6%) selected for the expressions systems at hand. The bottom left quadrant indicates the region of expression robustness and homogeneity.
(TIF)

S6 Fig Comparison of representative microcolonies from conducted microfluidic analyses, which differ in their *lacY* and *lacI* constitution. *lacY*^+^: *E*. *coli* BL21(DE3), *lacY*^-^: *E*. *coli* Tuner(DE3),—additional LacI: pRhotHi-2 expression vector, + additional LacI: pRhotHi-2-LacI expression vector. The white scale bar corresponds to 10 μm.
(TIF)

S1 Table Bacterial strains, plasmids and oligonucleotides used in this study. (PDF)

S2 Table Quantification of known inducing or repressing carbohydrates in different *E*. *coli* cultivation media. (PDF)
