# Supplementary material for: Comparative Single-Cell Analysis of Different E. coli Expression Systems during Microfluidic Cultivation
Source: PLoS One. 2016 Aug 15;11(8):e0160711. doi: 10.1371/journal.pone.0160711 (PMC4985164; doi:10.1371/journal.pone.0160711)
Supplement: S1 Appendix — Exact LB growth media recipes and quantification of galactose, lactose and glucose. (PDF) [file pone.0160711.s001.pdf]

Supporting Methods

LB Growth Media

For initial cultivations four different lysogeny broth (LB) cultivation media were employed that were constituted as follows: LB1 (25 g l^{-1}ready-to-use mix Luria/Miller; Carl Roth, Karlsruhe, Germany); LB2 (10 g l^{-1} tryptone/peptone from casein (Carl Roth, Karlsruhe, Germany), 10 g l^{-1} NaCl, 5 g l^{-1} yeast extract (type KAT, Ohly, Hamburg Germany); LB3 (10 g l^{-1} peptone from casein (Carl Roth, Karlsruhe, Germany), 10 g l^{-1} NaCl, 5 g l^{-1} yeast extract (Carl Roth, Karlsruhe, Germany); LB4 (10 g l^{-1} Bacto™ peptone (BD Biosciences, Franklin Lakes, NJ, USA), 10 g l^{-1} NaCl, 5 g l^{-1} Bacto™ yeast extract (BD Biosciences, Franklin Lakes, NJ, USA).

Quantification of galactose, lactose and glucose

Galactose and lactose were quantified *via* photometric detection of NADH using a β-galactosidase, galactose mutarotase and β-galactose dehydrogenase based enzyme assay (Rapid Kit K-LACGAR; Megazyme, Ireland). For glucose measurements a hexokinase/glucose-6-phosphate dehydrogenase based assay for NADPH detection was performed as previously described [58].

Supporting Reference

58. Richhardt J, Bringer S, Bott M. Role of the pentose phosphate pathway and the Entner-Doudoroff pathway in glucose metabolism of *Gluconobacter oxydans* 621H. Appl Microbiol Biotechnol. 2013; 97: 4315–23.
