# Supplementary figures and images for: Comparative Single-Cell Analysis of Different E. coli Expression Systems during Microfluidic Cultivation
Source: PLoS One. 2016 Aug 15;11(8):e0160711. doi: 10.1371/journal.pone.0160711 (PMC4985164; doi:10.1371/journal.pone.0160711)

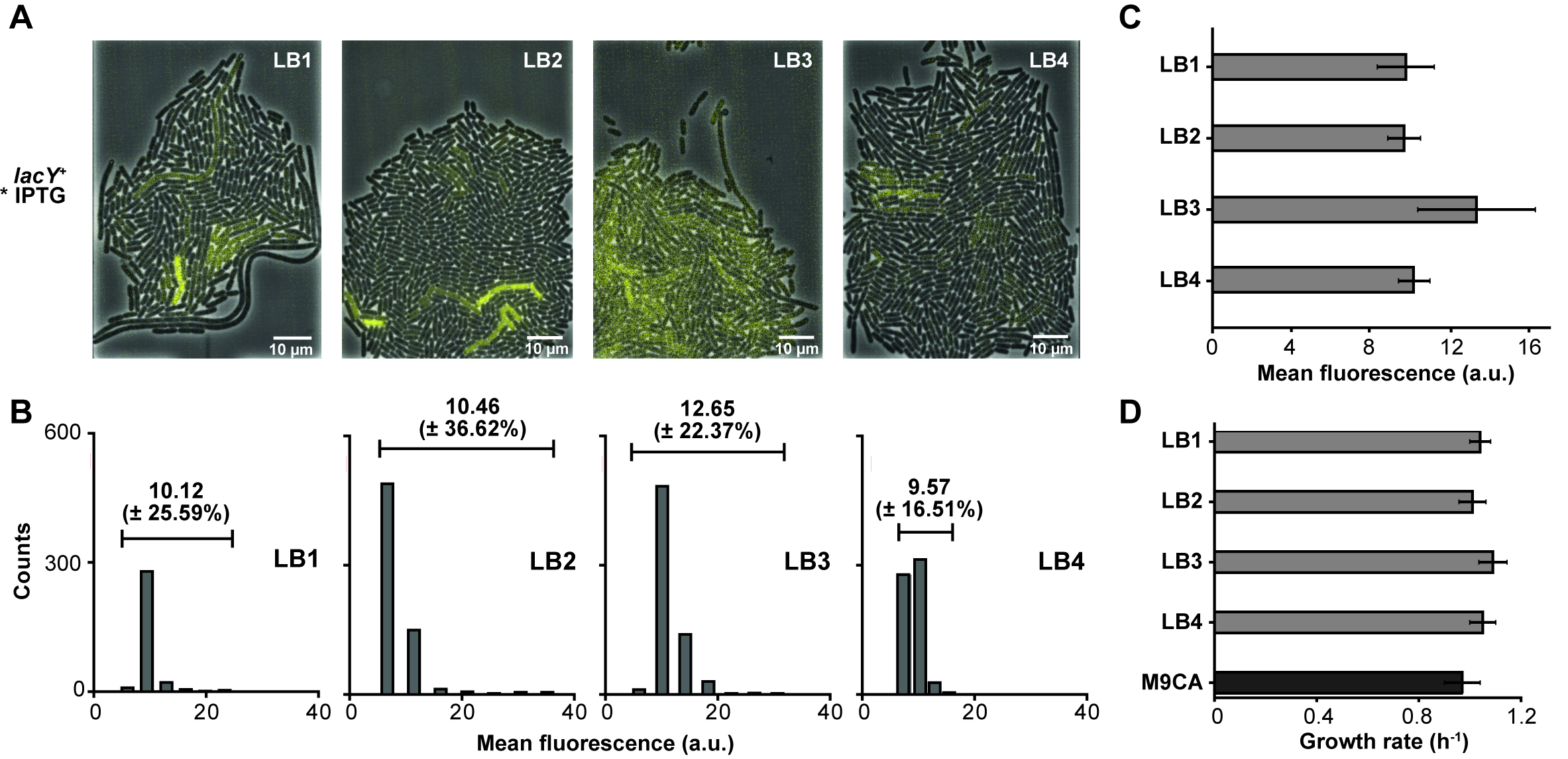

Supplement: S1 Fig — (A) Representative micro-colonies, weakly induced (2.5 μM) with IPTG after approximately 4 h of cultivation in four different LB media. (B) Mean fluorescence distribution for the representative microcolonies shown above. Mean values and coefficient of variations are plotted above the bar, indicating the complete spread. (C) Mean fluorescence for ten EYFP-expressing colonies cultivated in the four different media. (D) Comparison of maximum growth rates for non-induced cultivations in the different LB media (grey bars) with growth rates obtained for uninduced cultivation in the novel defined rich medium M9CA (dark grey bars). (TIF) [file pone.0160711.s002.tif]

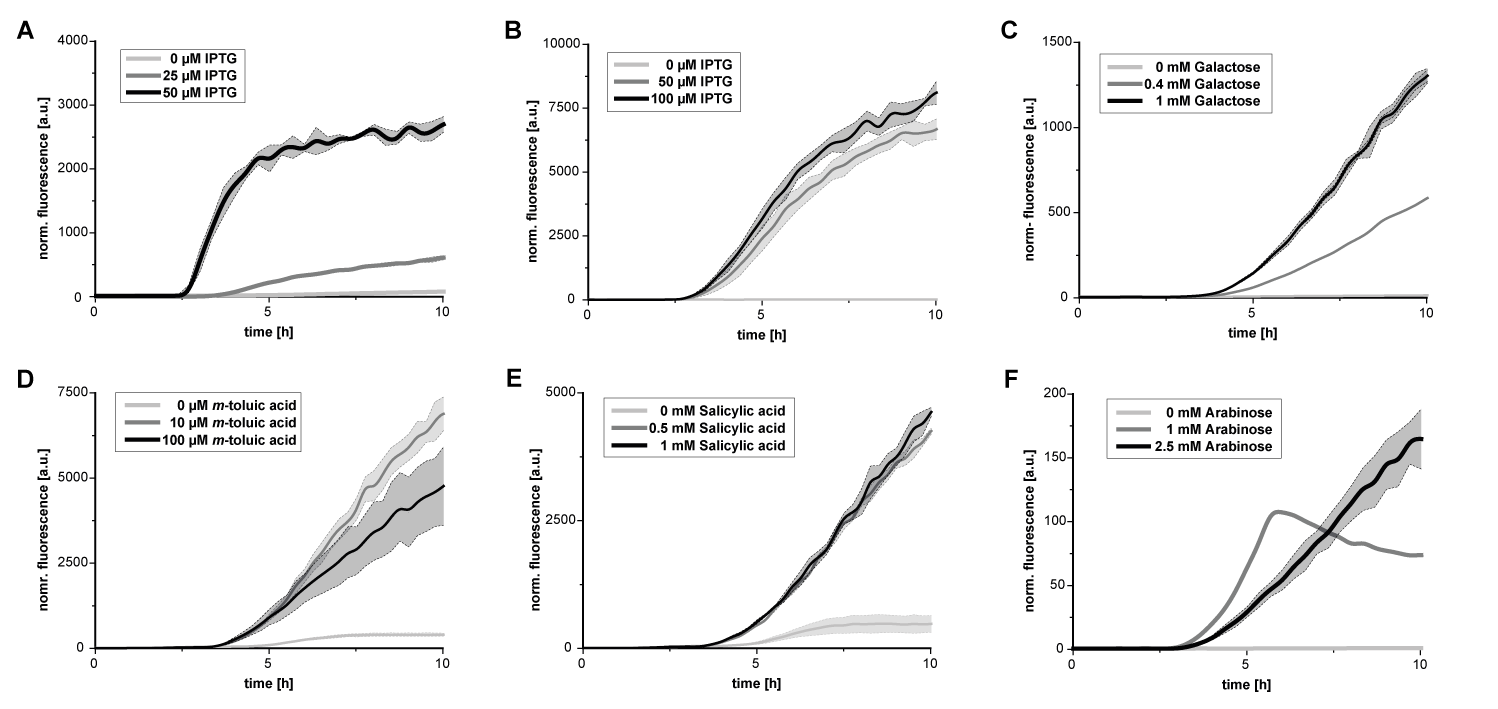

Supplement: S3 Fig — Expression response of the selected expression systems 1–6 (A-F) in a BioLector microbioreactor system (m2plabs, Germany) under constant monitoring of biomass accumulation and reporter fluorescence. Indicated fluorescence was biomass-normalized. Expression cultures were inoculated to cell densities corresponding to an optical density of 0.05 at 580 nm. Gene expression was induced when cell cultures reached the logarithmic growth phase (cell density of OD580 ~0.5). Cultures induced with 1 mM arabinose start to consume arabinose, while the are still growing, whereas induction with 2.5 mM arabinose leads to tremendous growth impairment and thus no arabinose consumption was observed during the observation period of 10 h. Expression cultures were performed at least in triplicates. Shaded areas indicate respective standard deviations. a.u.: arbitrary units. (TIF) [file pone.0160711.s004.tif]

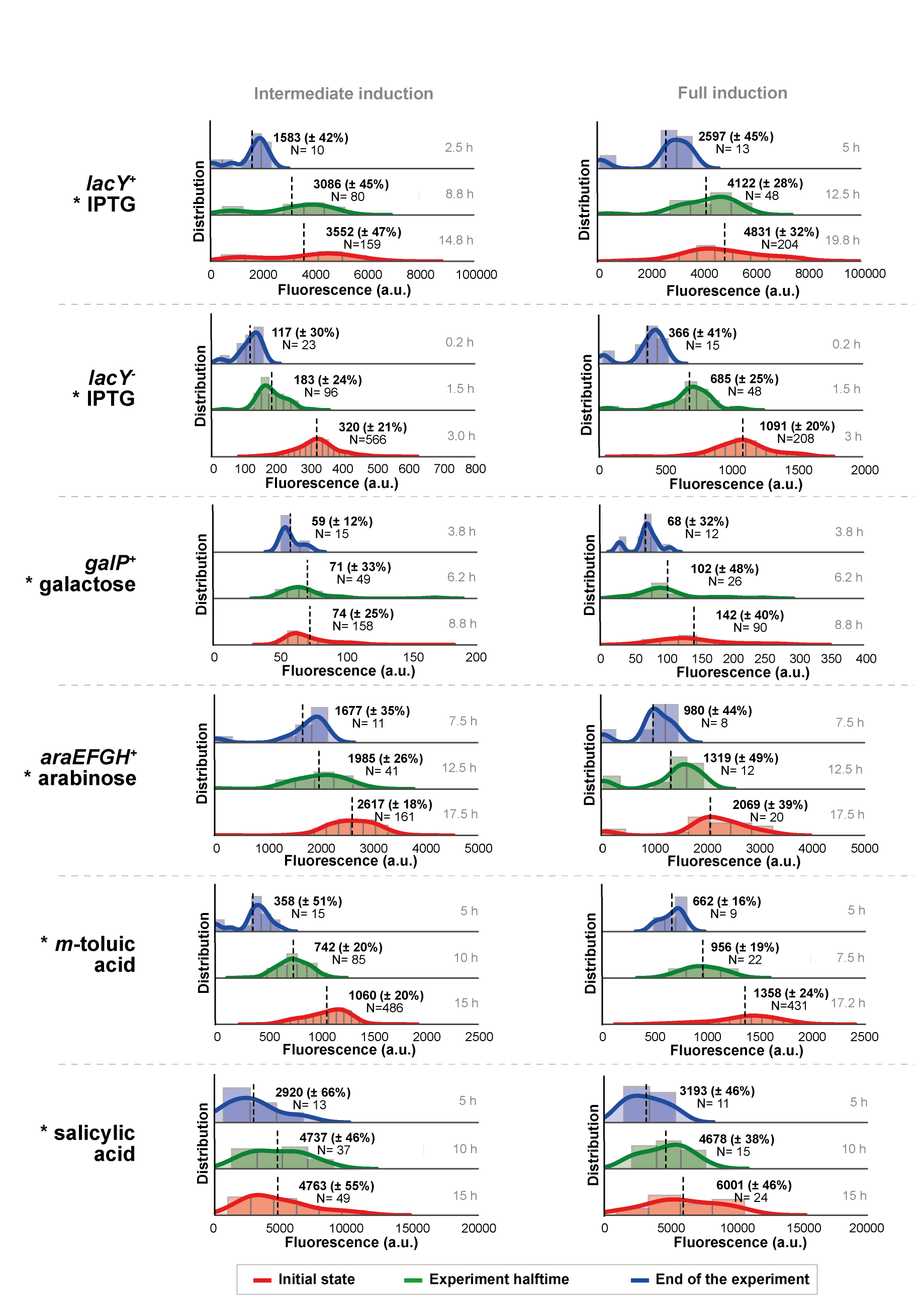

Supplement: S4 Fig — Histograms were plotted using single-cell fluorescence values obtained from representative populations at the initial (blue, N>8), intermediary (green, halftime of experiment) and end state (red, μmax ~ 0) of conducted microfluidic cultivation experiments. (TIF) [file pone.0160711.s005.tif]

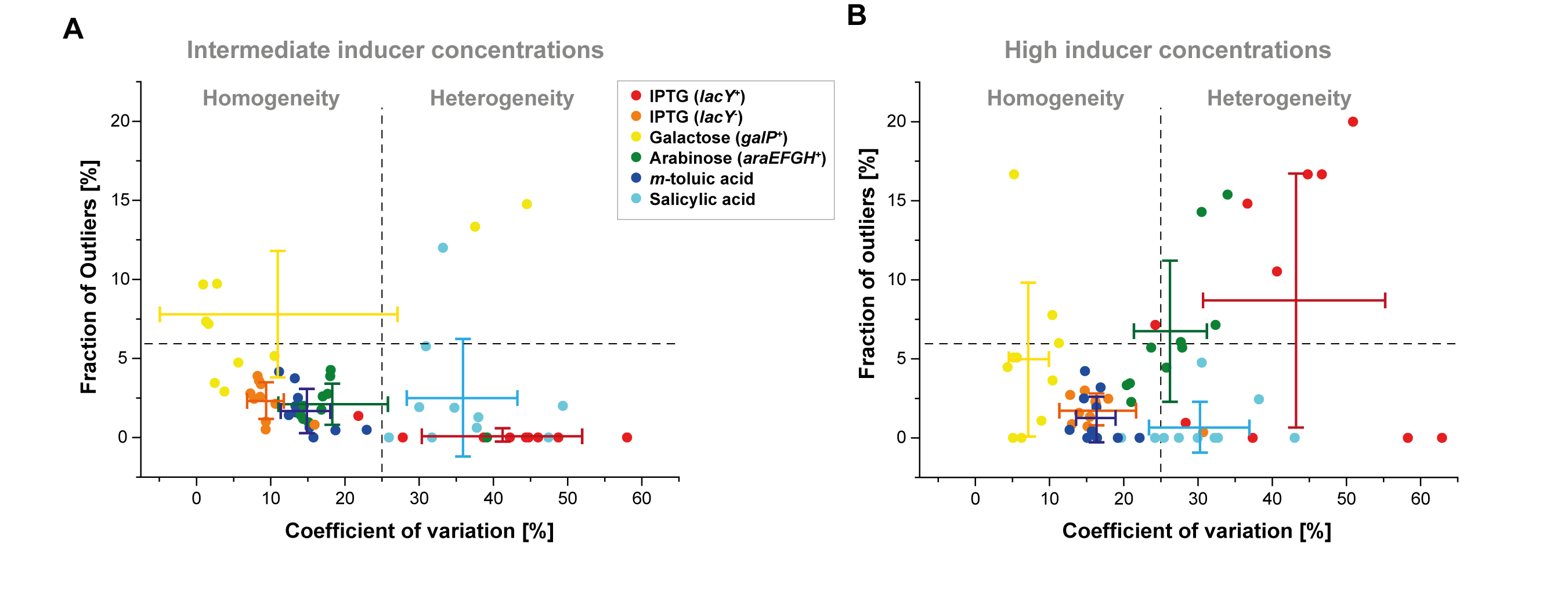

Supplement: S5 Fig — Percentaged coefficient of variation and fraction of outliers (outside the 1.5-fold IQR) are plotted as potential indicators of expression heterogeneity for ten individual microcolonies. Cross lines reveal respective means and standard deviations. Grey dotted lines show thresholds for expression heterogeneity (CV > 25%) or increased number of rare events (outliers > 6%) selected for the expressions systems at hand. The bottom left quadrant indicates the region of expression robustness and homogeneity. (TIF) [file pone.0160711.s006.tif]

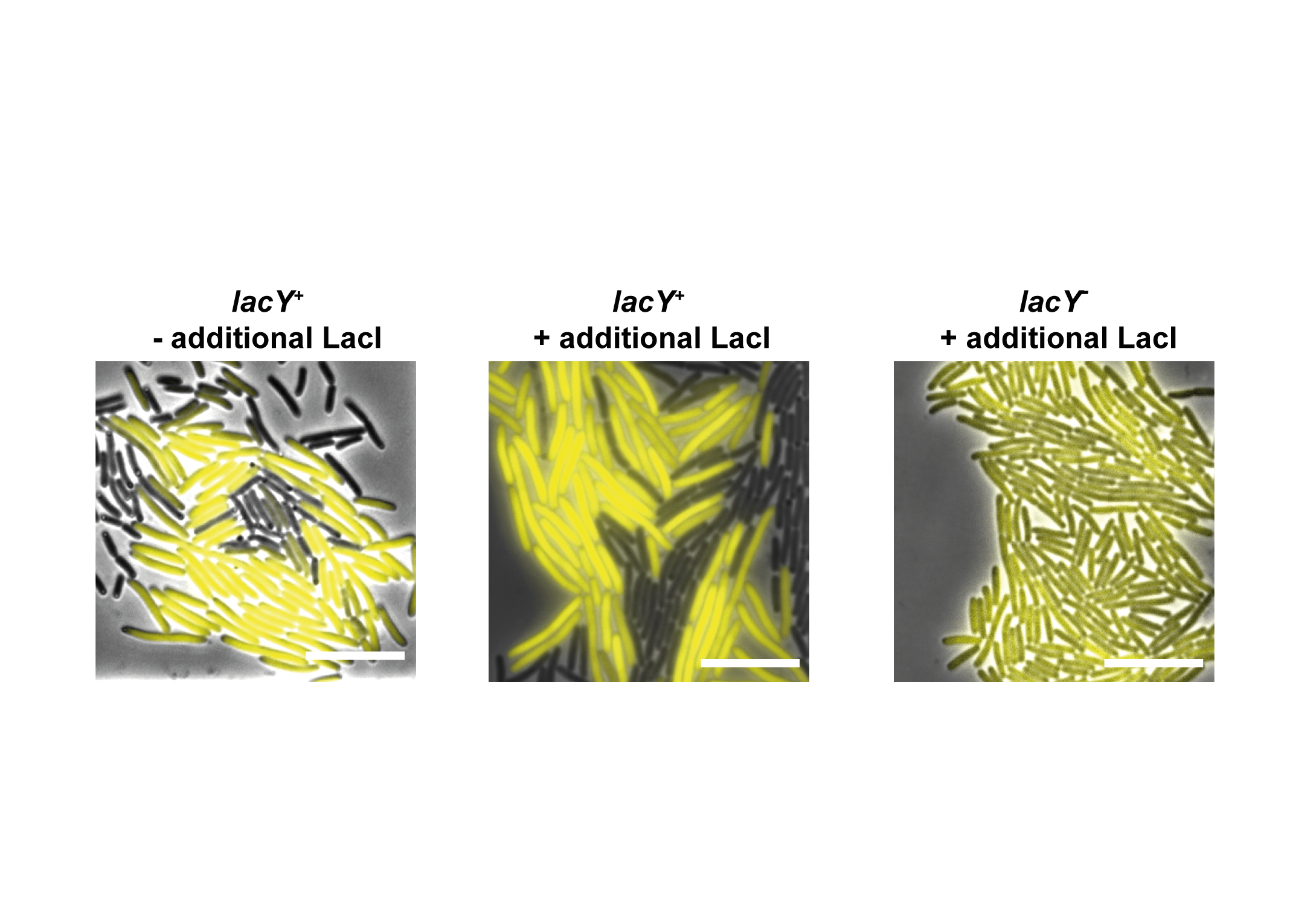

Supplement: S6 Fig — lacY+: E. coli BL21(DE3), lacY-: E. coli Tuner(DE3),—additional LacI: pRhotHi-2 expression vector, + additional LacI: pRhotHi-2-LacI expression vector. The white scale bar corresponds to 10 μm. (TIF) [file pone.0160711.s007.tif]
