# Supplementary material for: Comparative Single-Cell Analysis of Different E. coli Expression Systems during Microfluidic Cultivation
Source: PLoS One. 2016 Aug 15;11(8):e0160711. doi: 10.1371/journal.pone.0160711 (PMC4985164; doi:10.1371/journal.pone.0160711)
Supplement: S1 Table — (PDF) [file pone.0160711.s008.pdf]

S1 Table. Bacterial strains, plasmids and oligonucleotides used in this study.

Strain, plasmid, oligonucleotides	Relevant features, description or sequence*	Reference
**Strains**		
E. coli DH5α	*F⁻ Φ80lacZΔM15 Δ(lacZYA-argF) U169 recA1 endA1 hsdR17 (rk⁻, mk⁺) phoA supE44λ⁻ thi-1 gyrA96 relA1*	[56]
E. coli BL21(DE3)	*F⁻ ompT gal dcm lon hsdS$_B$(r$_B^-$ m$_B^-$)λ(DE3 [lacI lacUV5-T7 gene 1 ind1 sam7 nin5])*	[11]
E. coli Tuner(DE3)	*F⁻ ompT gal dcm lon hsdS$_B$ (r$_B^-$ m$_B^-$) lacY1(DE3)*	Novagen
**Plasmids**		
pRhotHi-2-EYFP	pBBR1-MCS-derivative, P_{T7}-lacO-MCS, Km^R, Cm^R, EYFP	[57]
pRhotHi-2-LacI-EYFP	pBBR1-MCS-derivative, P_{T7}-lacO-MCS, Km^R, Cm^R, pBBR22b-lacI, EYFP	[14]
pAra-GFP	pSBM2g backbone, araC, Km^R, P_{BAD}-GFPmut3	[48]
pSB-M117-2g	pMB1 replicon, xylS, P_{M117}-GFPmut3	[6]
pM117-R45T-GFP	pSB-M117-2g with R45T mutation of XylS	This work
**Oligonucleotides**		
1 (XylS_SalI_for)	Binds upstream of *Sal*I-site after *xylS*.<br>Sequence: 5'-GAGACACAACGTGGCTTTCC-3'	This work
2 (XylS_SacI_rev)	Binds upstream of *Sac*I-site in front of *xylS*.<br>Sequence: 5'- ATCGACTTGGCGCCTTTCTAC-3'	This work
3 (XylS_R45T_rev)	Binds within *xylS*. Mediates R45T point mutation.<br>Sequence: 5'- CAGGCAC̲GCTGCACCACAGAATC-3'	This work
4 (XylS_R45T_for)	Binds within *xylS*. Mediates R45T point mutation.<br>Sequence: 5'- GATTCTGTGGTGCAGCG̲TGCCTG-3'	This work

* Underlined sequence indicates point mutation used for XylS Mutagenesis (AGG → ACG).
