# Supplementary material for: Comparative Single-Cell Analysis of Different E. coli Expression Systems during Microfluidic Cultivation
Source: PLoS One. 2016 Aug 15;11(8):e0160711. doi: 10.1371/journal.pone.0160711 (PMC4985164; doi:10.1371/journal.pone.0160711)
Supplement: S2 Table — (PDF) [file pone.0160711.s009.pdf]

S2 Table. Quantification of known inducing or repressing carbohydrates in different *E. coli* cultivation media.

Medium	Reference	Glucose [mg l^{-1}]	Lactose [µg l^{-1}]	Galactose [µg l^{-1}]
**LB medium 1**	[48]	91.5 ± 1.3	5.3 ± 0.1	1.9 ± 0.3
**LB medium 2**	[14]	26.2 ± 0.7	5.3 ± 0.3	0.9 ± 0.3
**LB medium 3**	[59]	39.7 ± 1.9	6.4 ± 0.1	0.8 ± 0.1
**LB medium 4**	This study	52.1 ± 1.2	2.1 ± 0.3	n.d.
**M9CA medium**	This study	n.d.	n.d.	n.d.

n.d.: not detected
